# Nonlinear relationship between stress hyperglycemic ratio and prognosis in patients with cardiac surgery-related kidney injury: a retrospective cohort study

**DOI:** 10.1080/0886022X.2026.2613479

**Published:** 2026-01-28

**Authors:** Xiaopo Gao, Cheng Li, Yurou Wang, Jinlong Luo, Chengye Zhan

**Affiliations:** ^a^ Department of Emergency Medicine, Tongji Hospital, Tongji Medical College, Huazhong University of Science and Technology, Wuhan, China; ^b^ Department of Critical Care Medicine, Tongji Hospital, Tongji Medical College, Huazhong University of Science and Technology, Wuhan, China

**Keywords:** Stress hyperglycemia ratio, cardiac surgery, prognosis, MIMIC-IV

## Abstract

The prognostic role of the stress-induced hyperglycemia ratio (SHR) in patients with acute kidney injury related to cardiac surgery has not been fully explored. This study aims to examine the association between the SHR index and mortality in patients with cardiac surgery-associated acute kidney injury (CS-AKI). Data for this research were collected from the MIMIC database. This study investigated the relationship between SHR and prognosis of CS-AKI patients by survival analysis, restricted cubic lines, and subgroup analysis. In the final analysis, 3,249 patients were categorized into four groups based on the quartiles of the SHR. Multivariable Cox proportional hazards regression analysis demonstrated that patients in the highest quartile (Q4) had a significantly increased risk of mortality compared to those in the lower three quartiles (Q1–Q3) (*p* < 0.005). Receiver operating characteristic (ROC) curve analysis indicated a U-shaped relationship between SHR and patient mortality, with both low and high SHR values associated with increased risk. Incorporation of SHR into existing prognostic models (SHR+SAPS II, SHR+APACHE III, and SHR+SOFA) led to improved discriminative performance, as reflected by increased area under the curve (AUC) values. Additionally, the inclusion of SHR significantly enhanced model performance as demonstrated by net reclassification improvement (NRI) and integrated discrimination improvement (IDI) metrics (*p* < 0.046). The findings of this study indicate a U-shaped association between the SHR and prognosis in patients with CS-AKI. However, only elevated SHR values were independently associated with an increased risk of mortality after adjustment for confounding variables.

## Introduction

Acute kidney injury is one of the most common complications after cardiac surgery [1]. The incidence of cardiac surgery–associated acute kidney injury (CS-AKI) ranges from approximately 5 to 42%, with 1–5% of patients requiring renal replacement therapy (RRT) [2,3]. In particular, CS-AKI patients have mortality rates three to eight times higher than those without renal dysfunction, with mortality exceeding 50% in severe cases requiring renal replacement therapy (RRT) [4,5]. Thus, early identification of patients with CS-AKI is important for improving their prognosis.

Hyperglycemia has been observed to be a frequent occurrence in the perioperative setting, with prevalence rates of up to 40% in non-cardiac surgical procedures and as high as 80% in cardiac surgeries [6]. Hyperglycemia has been shown to be closely linked to elevated overall morbidity and death rates in hospitalized patients. It is particularly acknowledged as a substantial risk factor for postoperative complications [7–9]. Furthermore, postoperative hyperglycemia has been demonstrated to elevate the risk of systemic blood infections and acute kidney damage (AKI) [10]. Importantly, it was observed that patients undergoing major surgeries, even in cases of complete renal recovery, exhibited a significantly elevated risk of postoperative death [11]. In diabetic animal models, hyperglycemia induces reduced expression of silent information regulator 1 (SIRT1), leading to decreased autophagy and mitochondrial dysfunction, which in turn induces podocyte injury [12]. An animal investigation demonstrated that hyperglycemic rats displayed increased levels of hemorrhagic creatinine and more pronounced characteristics of acute tubular necrosis under ischemic conditions compared to normoglycemic rats. Nonetheless, the differences were not evident in normoglycemic rats subjected to ischemia [13].

SHR is a novel metric designed to quantify the acute glycemic response to physiological stress relative to an individual’s baseline glycemic status [14]. Unlike absolute blood glucose levels, SHR accounts for preexisting glycemic variability, making it a more precise indicator of stress-induced metabolic dysregulation. Mechanistically, stress-induced hyperglycemia is thought to contribute to renal damage through pathways such as endothelial dysfunction, increased oxidative stress, and pro-inflammatory cytokine release, all of which are implicated in the pathogenesis of CSA-AKI [15,16]. Recent research indicates that SHR may serve as an independent predictive marker across diverse clinical populations, including patients with sepsis and patients with severe cerebrovascular disease [17]. There is no compelling evidence to explicitly link SHR to CSA-AKI.

Therefore, the aim of this study was to evaluate the relationship between SHR and the prognosis of patients with CS-AKI. With this study, we hope to reduce the mortality of CS-AKI patients by providing more precise glucose control.

## Methods

### Data sources

The data utilized in the present research came from the MIMIC-IV v2.0 database. This database has detailed clinical data on over 250,000 hospitalizations, including demographics, laboratory values, prescription records, vital signs, and unstructured clinician notes. The person responsible for data extraction in this study has obtained access to this database (Approval ID: 66236335).

### Patients

Patients over 18 years of age who developed CS-AKI during ICU admission were included in this study. CS-AKI was defined as patients undergoing cardiac surgery identified by ICD procedure codes (Supplementary eTable 1) who subsequently developed AKI according to KDIGO serum creatinine criteria (increase ≥0.3 mg/dL within 48 h or ≥1.5 times baseline within 7 days after surgery) [18]. Additionally, the distribution of patients with acute kidney injury following cardiac surgery has been detailed in the supplementary document (Supplementary eTable 2). For patients with multiple ICU admissions after their first cardiac surgery, only the first admission was retained. Baseline serum creatinine (SCr) was defined as the lowest value within 7 days prior to hospitalization. For patients without a pre-hospital SCr record, the first measurement on admission was used as the baseline.

**Figure 1.**
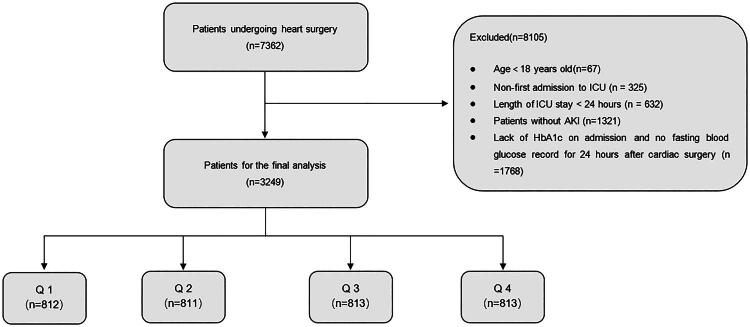
Flowchart of patient selection.

The final number of patients included in this study was 3,249 (Figure 1), and the exclusion criteria were (1) individuals not admitted to the ICU for the first time following their initial cardiac surgery, (2) patients who lacked glycated hemoglobin and blood glucose on admission, and (3) patients who were in the ICU for under 24 h. They were subsequently categorized into four groups according to SHR quartiles: Q1 (*n* = 812), Q2 (*n* = 811), Q3 (*n* = 813), and Q4 (*n* = 813).

### Data collection and management of missing values

Navicat Premium (version 17.0.3) with SQL was used to extract the data. Variables included were (1) demographics: age and sex; (2) laboratory tests: including blood glucose, glycated hemoglobin, white blood cell count, creatinine, urea nitrogen, platelet count, serum sodium, serum potassium, and hemoglobin; Among these, blood glucose refers to the first glucose value within 24 h after ICU admission; HbA1c represents the most recent HbA1c measurement taken within the 3 months prior to ICU admission; if multiple results exist, the one closest to the ICU admission date is selected; (3) baseline characteristics: systolic blood pressure, diastolic blood pressure, heart rate, and respiratory rate; (4) comorbidities: acute myocardial infarction, chronic pulmonary disease, diabetes mellitus, and malignancy, and congestive heart failure; (5) Critical care scores: including the APACHE III, SAPS II, and SOFA; (6) Surgery type: CABG only, valve only, combined (CABG+valve), others. In addition, we managed missing values, and for variables with missing values <20% (serum sodium (1.0%), serum potassium (0.8%), and hemoglobin (3.8%)), multiple imputation by chained equations (MICE) was applied, including all covariates, SHR, and mortality outcomes as predictors. Five imputed datasets were generated with 50 iterations each, and convergence was verified by trace plots. Results were pooled using Rubin’s rules. Covariates with missing values >20% (height (95%)) will not be included in subsequent studies. Additionally, we conducted a sensitivity analysis in a subgroup of patients with height and BMI data (*n* = 152). The baseline table was consistent with the primary analysis. Due to the small number of deaths at the endpoint, we were unable to further explore the relationship with the outcome (Supplementary eTables 3 and 4).

## Calculation of SHR and outcome

SHR = glucose (mg/dl)/(28.7 × HbA1c (%)-46.7) [19]. This study primarily aimed to assess all-cause mortality within 30 days, while also considering all-cause mortality at 90 and 360 days as secondary outcomes.

### Statistical analysis

Continuous variables were first tested for normality. Variables that did not meet the normal distribution are represented by medians (interquartile range). For continuous variables, *t*-tests or ANOVA were employed, and categorical variables were assessed using the chi-square test or Fisher’s exact test. We conducted an analysis of stratified SHR using Kaplan-Meier (KM) curves to assess the incidence of outcomes. In this study, three COX regression models were developed: model 1 accounted for demographic variables, model 2 included adjustments for laboratory indicators, and model 3 added comorbidities to the adjustments made in model 2. ROC curve analysis was used to predict mortality. And combined with critical care scores to predict mortality, the new model was evaluated by NRI, IDI, and calibration curves. In addition, we examined the potential nonlinear association between SHR and patient prognosis using RCS. Stratified analyses were performed according to sex, age, and complications. Furthermore, the developed models performed covariance analysis of the included covariates, and those with variance inflation factors exceeding 5 were removed (Supplementary eTable 5). All analyses covered in this study were performed by using R software (4.4.2).

## Results

### Baseline characteristics

In the MIMIC database, we identified 9,546 patients hospitalized in the ICU after undergoing cardiac surgery. The study finally included 3,249 patients who satisfied the criteria and were divided into four groups according to SHR quartiles (Figure 1). In terms of baseline features, patients in the Q4 group presented with lower systolic blood pressure (median (IQR): 111 (99.0–122.0) mmHg) and diastolic blood pressure (median (IQR): 57.0 (50.0–65.0) mmHg) along with higher serum potassium levels (median (IQR): 4.6 (4.0–5.1) mg/dL), compared to the Q1–Q3 groups (Table 1). Notably, the Q4 group demonstrated a significantly greater proportion of patients with acute kidney injury (AKI) stage 3 (*n* = 5.90%) and those requiring continuous renal replacement therapy (CRRT) (*n* = 5.17%) in comparison with the Q1–Q3 groups. Furthermore, the Q4 group exhibited significantly higher disease severity, with SOFA scores of 6 (IQR: 4–9) and Acute Physiology and Chronic Health Evaluation III (APACHE III) scores of 37.0 (IQR: 29.0–49.0), both of which were significantly higher than those of the other cohorts (*p* < 0.001).

**Table 1.** Baseline characteristics.

Variables	Total (*n* = 3,249)	Q1 *N* = 812 (SHR ≤ 1.19)	Q2 *N* = 811 (1.19 < SHR ≤ 1.39)	Q3 *N* = 813 (1.39 < SHR ≤ 1.61)	Q4 *N* = 813 (SHR > 1.61)	*p*
Age (years)	69.40 ± 11.05	68.91 ± 11.23	70.29 ± 10.19	69.68 ± 10.84	68.71 ± 11.83	0.002
Sex, *n* (%)						0.622
F	949 (29.21)	234 (28.82)	225 (27.74)	249 (30.63)	241 (29.64)	
M	2,300 (70.79)	578 (71.18)	586 (72.26)	564 (69.37)	572 (70.36)	
Initial vital signs						
SBP (mmHg)	112.00 (101.00, 124.00)	114.00 (102.75, 125.00)	112.00 (102.00, 124.00)	112.00 (101.00, 123.00)	111.00 (99.00, 122.00)	0.006
DBP (mmHg)	58.00 (51.00, 65.00)	58.00 (52.00, 66.00)	58.00 (51.00, 65.00)	58.00 (52.00, 65.00)	57.00 (50.00, 65.00)	0.219
RR (times/min)	16.00 (14.00, 18.00)	16.00 (14.00, 18.00)	16.00 (14.00, 16.00)	16.00 (14.00, 18.00)	16.00 (14.00, 18.00)	<0.001
HR (breaths/min)	80.00 (75.00, 86.00)	80.00 (74.00, 85.00)	80.00 (74.00, 85.00)	80.00 (74.00, 85.00)	80.00 (76.00, 88.00)	<0.001
Initial laboratory results						
WBC (×10^9^/L)	16.00 (12.60, 20.10)	15.00 (11.70, 18.80)	15.10 (12.30, 19.00)	16.40 (13.10, 20.20)	17.70 (13.80, 22.30)	<0.001
Platelet (×10^9^/L)	143.00 (114.00, 179.00)	146.00 (118.00, 183.00)	142.00 (112.00, 177.00)	143.00 (114.00, 181.00)	140.00 (110.00, 175.00)	0.01
HbA1c, %	5.90 (5.50, 6.70)	7.40 (6.20, 8.80)	6.00 (5.70, 6.60)	5.70 (5.40, 6.20)	5.40 (5.20, 5.80)	<0.001
Hemoglobin (g/dl)	9.90 (8.30, 11.40)	10.00 (8.30, 11.40)	10.00 (8.40, 11.50)	10.00 (8.50, 11.40)	9.60 (8.00, 11.20)	0.022
Glucose (mg/dl)	177.00 (158.00, 203.00)	168.00 (141.00, 200.00)	162.00 (150.50, 182.00)	175.00 (162.00, 193.00)	201.00 (183.00, 226.00)	<0.001
Neutrophils (×10^9^/L)	9.59 (6.93, 12.83)	8.56 (6.16, 11.03)	8.98 (6.49, 11.76)	9.77 (7.09, 12.91)	11.13 (8.34, 15.00)	<0.001
Sodium Idx1, mmol/L	135.00 (134.00, 137.00)	135.00 (134.00, 137.00)	135.00 (134.00, 137.00)	135.00 (134.00, 137.00)	135.00 (134.00, 137.00)	0.45
Potassium Idx1, mmol/L	4.50 (4.00, 5.00)	4.40 (4.00, 4.80)	4.50 (4.00, 5.00)	4.50 (4.10, 5.00)	4.60 (4.00, 5.10)	<0.001
Creatinine (mg/dl)	1.00 (0.90, 1.30)	1.10 (0.90, 1.40)	1.00 (0.80, 1.30)	1.00 (0.80, 1.30)	1.10 (0.90, 1.40)	<0.001
Glucose (mg/dl)	177.00 (158.00, 203.00)	168.00 (141.00, 200.00)	162.00 (150.50, 182.00)	175.00 (162.00, 193.00)	201.00 (183.00, 226.00)	<0.001
BUN (mg/dl)	16.00 (13.00, 21.00)	17.00 (13.00, 23.00)	16.00 (12.00, 20.50)	16.00 (13.00, 20.00)	16.00 (13.00, 21.00)	<0.001
Surgery type						<0.001
CABG only	1, 130 (34.78)	355 (43.72)	317 (39.09)	254 (31.24)	204 (25.09)	
Valve surgery only	584 (17.97)	102 (12.56)	120 (14.80)	163 (20.05)	199 (24.48)	
Combined (CABG+valve)	345 (10.62)	68 (8.37)	80 (9.86)	90 (11.07)	107 (13.16)	
Others	1, 190 (36.63)	287 (35.34)	294 (36.25)	306 (37.64)	303 (37.27)	
Disease severity scores						
SAPS II	37.00 (31.00, 44.00)	36.00 (31.00, 43.00)	37.00 (31.00, 44.50)	37.00 (31.00, 44.00)	38.00 (31.00, 45.00)	0.125
APACHE III	35.00 (27.00, 46.00)	35.00 (28.00, 45.00)	34.00 (27.00, 46.00)	34.00 (26.00, 46.00)	37.00 (29.00, 49.00)	<0.001
SOFA	3.00 (1.00, 5.00)	3.00 (1.00, 4.00)	3.00 (1.00, 4.00)	3.00 (1.00, 5.00)	3.00 (2.00, 5.00)	<0.001
Aki stage, *n* (%)						<0.001
Stage 1	2, 515 (77.41)	614 (75.62)	649 (80.02)	640 (78.72)	612 (75.28)	
Stage 2	622 (19.14)	171 (21.06)	141 (17.39)	157 (19.31)	153 (18.82)	
Stage 3	112 (3.45)	27 (3.33)	21 (2.59)	16 (1.97)	48 (5.90)	
Diabetes, *n* (%)						
Chronic obstructive pulmonary disease, *n* (%)	695 (21.39)	187 (23.03)	183 (22.56)	157 (19.31)	168 (20.66)	0.23
Congestive heart failure, *n* (%)	1, 166 (35.89)	301 (37.07)	241 (29.72)	290 (35.67)	334 (41.08)	<0.001
Diabetes mellitus, *n* (%)	1, 284 (39.52)	568 (69.95)	321 (39.58)	224 (27.55)	171 (21.03)	<0.001
Myocardial infarct, *n* (%)	1, 284 (39.52)	370 (45.57)	314 (38.72)	289 (35.55)	311 (38.25)	<0.001
Malignant cancer, *n* (%)	81 (2.49)	22 (2.71)	19 (2.34)	24 (2.95)	16 (1.97)	0.602
Clinical outcomes, *n* (%)						
30-day mortality (%)	90 (2.77)	15 (1.85)	16 (1.97)	18 (2.21)	41 (5.04)	<0.001
90-day mortality (%)	137 (4.22)	24 (2.96)	26 (3.21)	29 (3.57)	58 (7.13)	<0.001
360-day mortality (%)	238 (7.33)	57 (7.02)	47 (5.80)	48 (5.90)	86 (10.58)	<0.001

*Note*: SBP, systolic blood pressure; DBP, diastolic blood pressure; RR, respiratory rate; HR, heart rate; WBC, white blood cell; BUN, blood urea nitrogen; HbA1c, glycosylated hemoglobin; SAPS II, Simplified Acute Physiology Score; APACHE III, Acute Physiology and Chronic Health Evaluation III; SOFA, Sequential Organ Failure Assessment; AKI, acute kidney injury; CABG, coronary artery bypass grafting.

### Relationship between SHR and clinical outcomes

Mortality rates exhibited considerable variation among the groups, with the Q4 group demonstrating the greatest 30-day (5.04%), 90-day (7.13%), and 360-day (10.58%) mortality rates among hospitalized patients. Univariable Cox regression analysis revealed that patients in the Q4 group (SHR ≥1.61) displayed significantly elevated death rates at 30-day, 90-day, and 360-day intervals compared to the Q1–Q3 groups. However, no notable differences were found between Q1 and Q2 or Q1 and Q3 (*p* > 0.05). The data indicate that a postoperative SHR ≥ 1.61 correlates with a substantially elevated mortality risk at all three-time intervals as compared to individuals with SHR < 1.61. Furthermore, the prognostic impact of SHR was evaluated through three sequentially adjusted Cox models: Model 1 incorporated demographic covariates; Model 2 added biochemical parameters to the Model 1 covariates; and Model 3 further adjusted for comorbidities based on Model 2. Multivariable Cox proportional hazards regression analyses revealed that elevated stress hyperglycemia ratio (SHR) in quartile 4 (Q4) remained an independent predictor for mortality among patients with CS-AKI (Table 2). In addition, considering that diabetes mellitus may have an impact on the results, further interaction analysis of SHR with diabetes mellitus and stratified multivariate Cox regression between diabetic and nondiabetic groups were performed. The results showed a significant interaction (interaction *p* < 0.005) (Supplementary eTable 6). Stratified multivariate Cox regression analyses showed that in diabetic patients, the highest quartile (Q4) of SHR was associated with a significantly higher risk of 30-, 90-, and 360-day mortality. In nondiabetic patients, SHR was mainly associated with short-term mortality, and Q4 was significantly associated with 30-day mortality in all three models, whereas no significant association was seen for 360-day mortality (Supplementary eTables 7 and 8).

**Table 2.** Multivariate cox regression analysis of 30-, 90- and 360-day mortality in patients with CS-AKI.

		Model 1			Model 2			Model 3		
Variable		HR	95%CI	*p*-Value	HR	95%CI	*p*-Value	HR	95%CI	*p*-Value
SHR quartiles										
	30-day mortality									
	Q1	1			1			1		
	Q2	1.04	0.60–1.78	0.899	0.98	0.48–2.01	0.949	1.18	0.57–2.45	0.651
	Q3	1.27	0.76–2.14	0.358	1.04	0.51–2.10	0.921	1.39	0.67–2.89	0.376
	Q4	2.79	1.84–4.22	<0.001	2.3	1.25–4.23	<0.001	3.44	1.81–5.04	<0.001
	90-day mortality									
	Q1	1			1			1		
	Q2	1.05	0.68–1.60	0.835	1.14	0.64–2.04	0.655	1.3	0.72–2.34	0.39
	Q3	1.26	0.84–1.90	0.265	1.23	0.69–2.18	0.476	1.6	0.89–2.88	0.117
	Q4	2.35	1.67–3.30	<0.001	2.23	1.34–3.73	0.002	3.33	1.95–5.70	<0.001
	360-day mortality									
	Q1	1			1			1		
	Q2	0.82	0.60–1.13	0.226	0.81	0.54–1.21	0.302	0.93	0.62–1.40	0.741
	Q3	0.87	0.63–1.19	0.37	0.89	0.60–1.33	0.571	1.02	0.68–1.55	0.912
	Q4	1.45	1.11–1.89	0.006	1.59	1.11–2.26	0.010	1.82	1.25–2.65	0.002

Inclusion variables: model 1: Sex, Age, Systolic blood pressure, Heart rate, Respiratory rate; model 2: model 1 + White blood cell count, Platelet count, Creatinine, Blood urea nitrogen, Hemoglobin, Serum sodium, Serum potassium model 3: model 2 + Malignancy, Congestive heart failure, COPD, Diabetes mellitus, Acute myocardial infarction.

Furthermore, Kaplan-Meier survival analyses demonstrated that patients in the Q4 group exhibited a markedly elevated mortality risk relative to the other three groups (log-rank *p* < 0.001) (Figure 2).

Figure 2.Kaplan-Meier curves for all-cause mortality.

**Figure:**
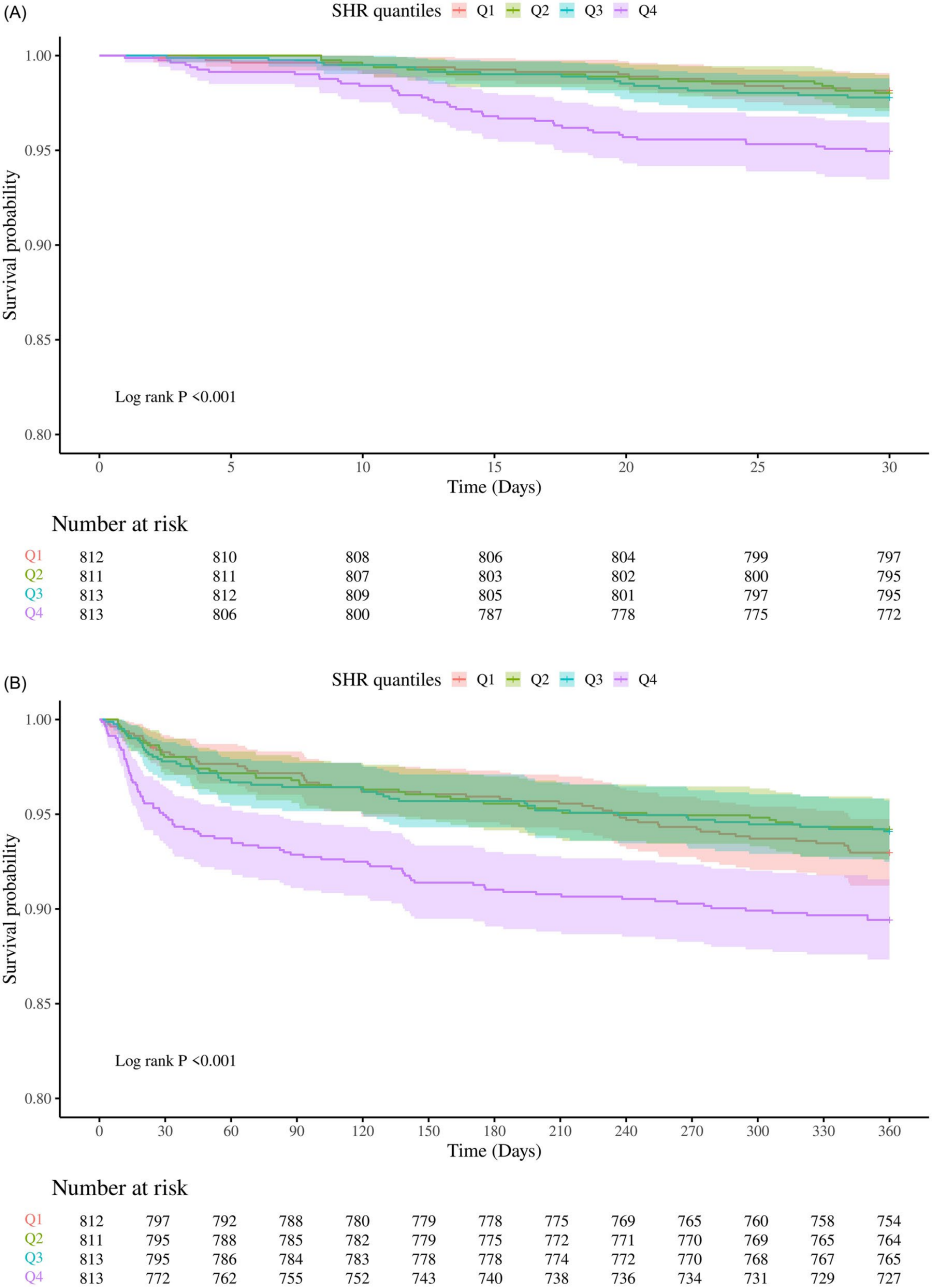

**Figure:**
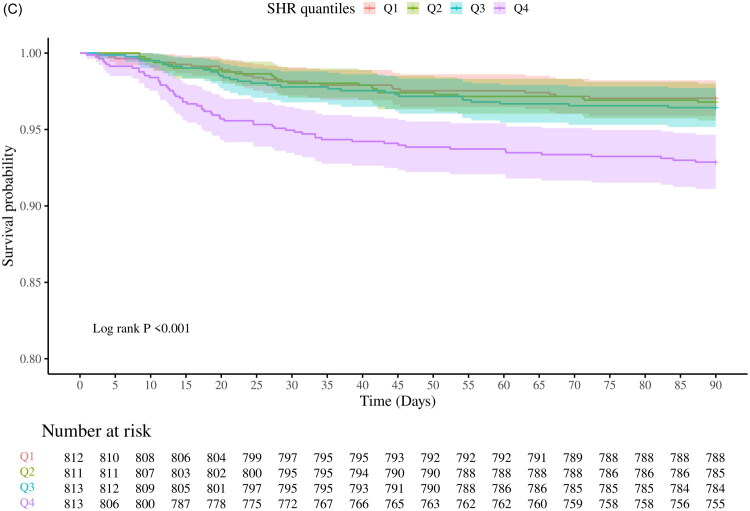

### Predictive value and incremental effect of SHR

**Figure 3.**
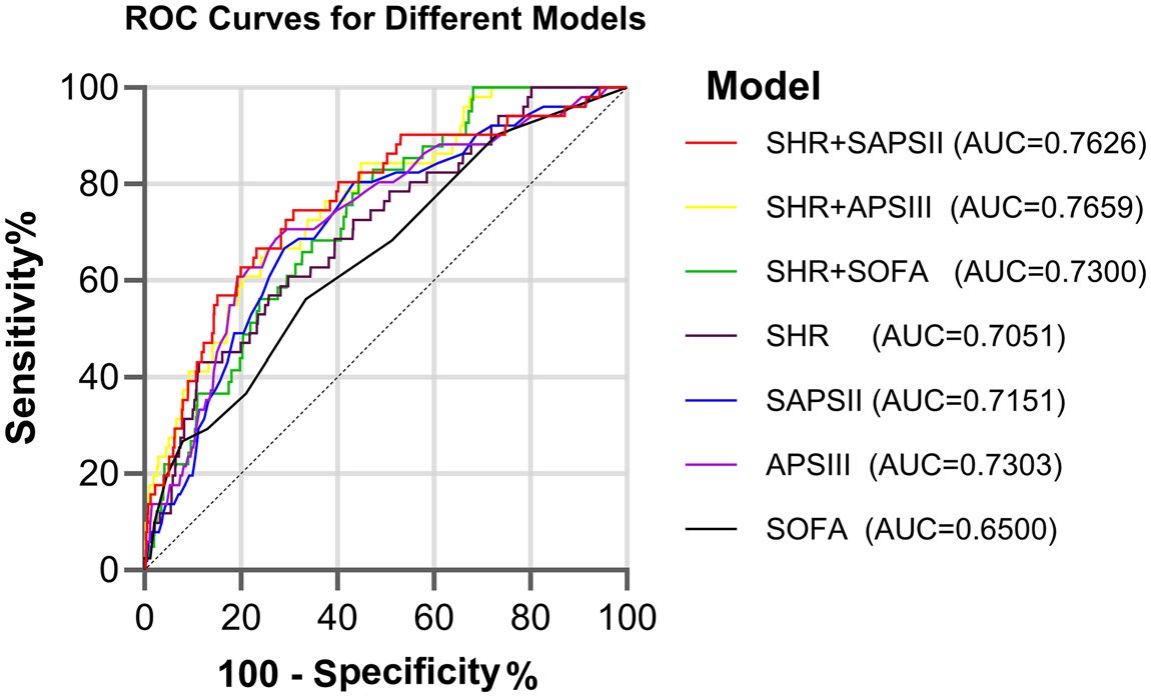
ROC curve analysis of the incremental effect of SHR on 30-day all-cause mortality.

We established ROC curves to assess the predictive efficacy of the SHR for 30-day mortality. Furthermore, the incremental prognostic utility of SHR was examined by integrating it with established clinical scores, including the APACHE III, SAPSII, and SOFA (Figure 3). The incorporation of the SHR into established scoring systems significantly enhanced their prognostic performance. The AUC increased from 0.715 (95% CI: 0.646–0.782) to 0.762 (95% CI: 0.742–0.856) for SAPS II, from 0.730 to 0.766 for APACHE III, and from 0.650 to 0.730 for SOFA (*p* < 0.001 for all comparisons). Further evaluation using NRI and IDI confirmed the incremental predictive value of SHR: SAPS II + SHR: NRI = 12.98% (95% CI: 3.52–22.43; *p* = 0.007), IDI = 2.43% (95% CI: 0.91–3.95; *p* = 0.002), APACHE III + SHR: NRI = 12.91% (95% CI: 3.46–22.36; *p* = 0.008), IDI = 2.51% (95% CI: 0.82–4.19; *p* = 0.003). SOFA + SHR: NRI = 7.15% (95% CI: −0.23–14.54; *p* = 0.058), IDI = 1.72% (95% CI: 0.53–2.90; *p* = 0.046) (Table 3). In addition, we performed calibration analysis, including plotting the calibration curve based on bootstrap resampling (*B* = 500) and performing the Hoser-Lemeshow goodness-of-fit test. The results showed that the calibration curve predictions were highly consistent with the actual, and none of the Hosmer-Lemeshow tests were statistically different (*p* > 0.05), suggesting that the model has excellent calibration and overall accuracy (Supplementary eFig.1–eFig.3).

**Table 3.** Enhancement in discrimination and risk reclassification for 30-day mortality following the inclusion of SHR.

	NRI (%) (95%CI)	*p*	IDI (%) (95%CI)	*p*
SAPSII	Ref	Ref	Ref	Ref
SAPSII+SHR	12.98 (3.52–22.43)	0.007	2.43 (0.91–3.95)	0.002
APACHE III	Ref	Ref	Ref	Ref
APACHE III+SHR	12.91 (3.46–22.36)	0.008	2.51 (0.82–4.19)	0.003
SOFA	Ref	Ref	Ref	Ref
SOFA+SHR	7.15 (−0.23–14.54)	0.058	1.72 (0.53–2.9)	0.046

*Note*: SHR, the stress-induced hyperglycemia ratio; SAPS II, Simplified Acute Physiology Score; APACHE III, Acute Physiology and Chronic Health Evaluation III; SOFA, Sequential Organ Failure Assessment.

### Nonlinear relationship between SHR and patient prognosis

The RCS results showed a significant nonlinear and U-shaped relationship between SHR and 30-day, 90-day, and 360-day mortality rates in patients with CS-AKI. The lowest risk point was identified at SHR = 1.39 (Figure 4). Below this threshold (SHR < 1.39), mortality risk decreased with increasing SHR, whereas above this threshold (SHR ≥ 1.39), mortality risk escalated significantly as SHR increased.

Figure 4.RCS analysis of SHR index and mortality in patients with CS-AKI.

**Figure:**
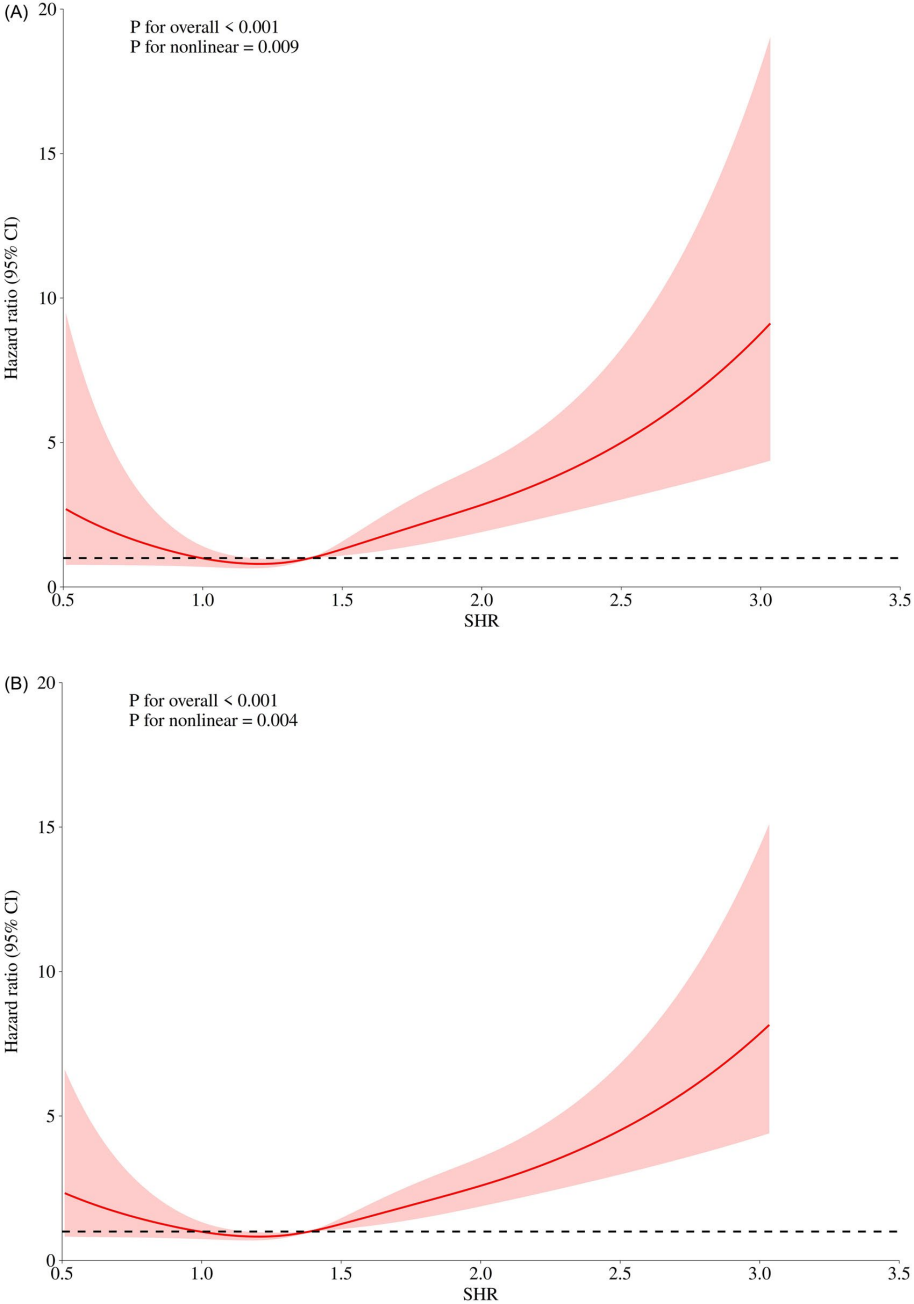

**Figure:**
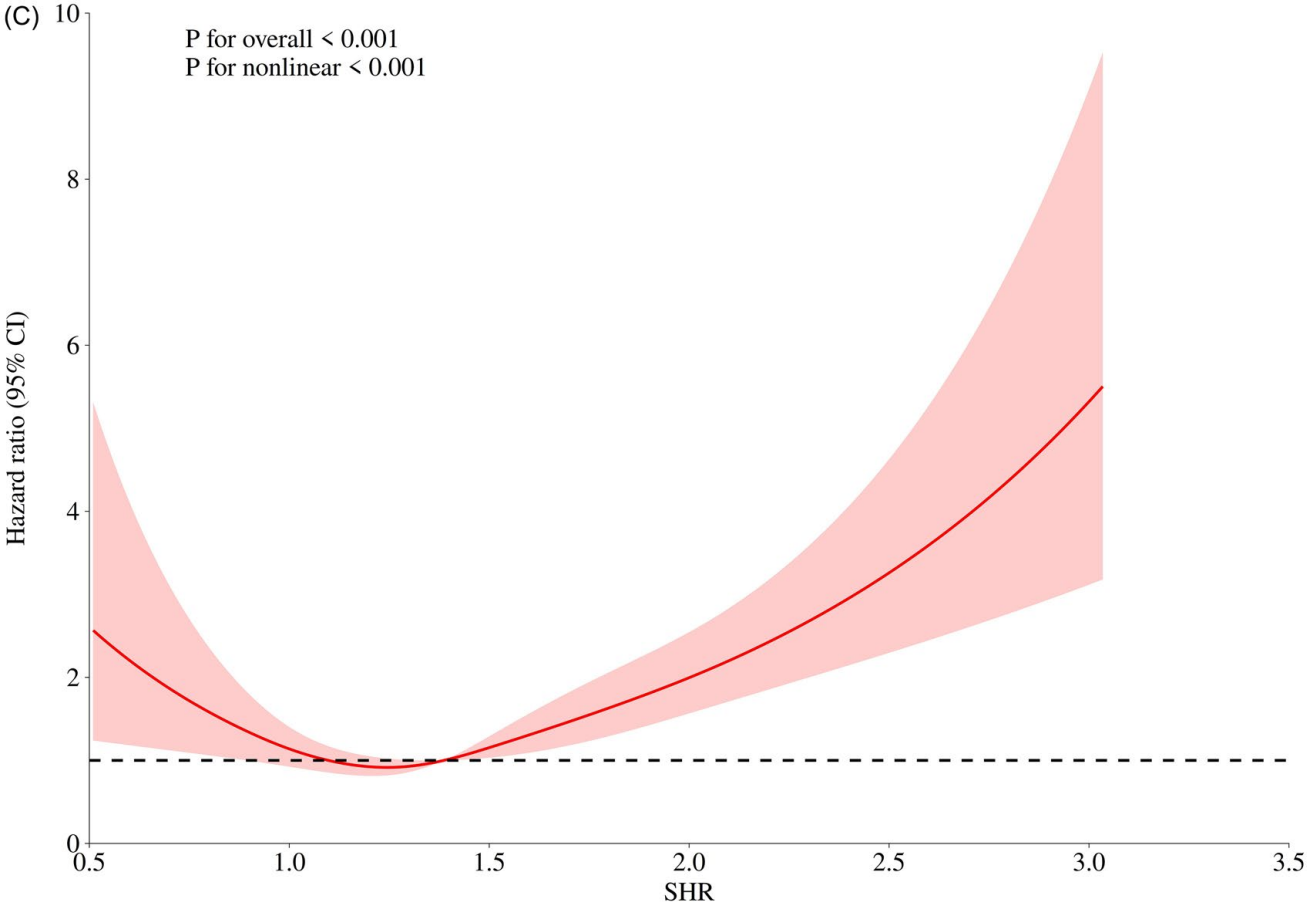

### Subgroup analysis

**Figure 5.**
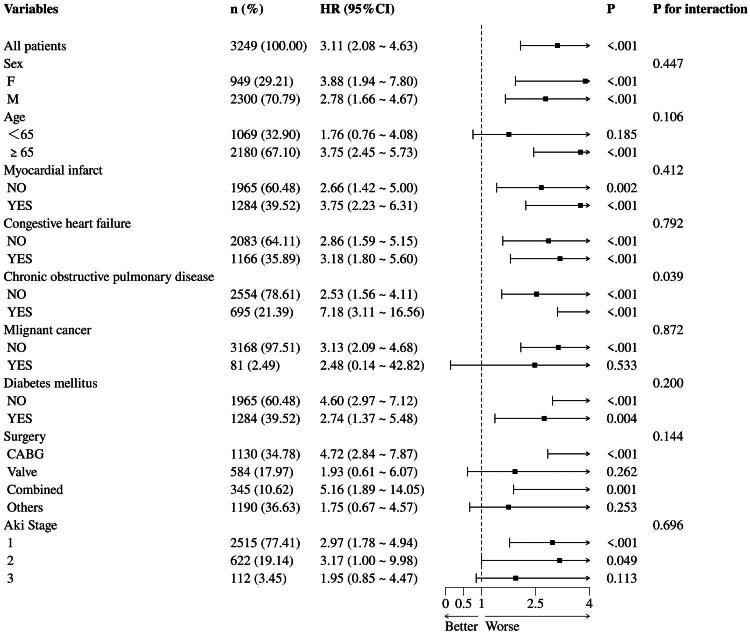
Effect of SHR index on 30-day mortality in different subgroups.

In the overall cohort (*n* = 3,249), the SHR exhibited a significant correlation with 30-day mortality. Subgroup analyses consistently showed elevated risk (HR > 1) in all subgroups, but with varying degrees of impact (Figure 5). However, patients with combined malignancy had a lower HR than patients without combined malignancy, and the reason for this result may be explained by the smaller number of patients with combined malignancy (2.49%). The interaction *p*-value was significantly significant only in the subgroup with COPD (*p* = 0.039), indicating that COPD status significantly altered the strength of the correlation with SHR and mortality. Patients with comorbid COPD had HR = 7.18 (*p* < 0.001), which was notably elevated in comparison to patients without comorbid COPD (HR = 2.53). The presence of COPD increased the correlation between SHR and 30-day mortality (HR increased from 2.53 to 7.18). No associations were detected for the other subgroups (*p*-value > 0.05 for all interactions).

## Discussion

Our study wanted to examine the correlation between SHR and prognosis in patients with CS-AKI. This research indicates that a higher SHR is an independent predictor of all-cause mortality in patients with CS-AKI. Findings reveal that patients in the highest SHR quartile (Q4, SHR ≥ 1.61) even after rigorous adjustment for demographics, comorbidities, and biochemical parameters. Additionally, incorporation of the SHR into established prognostic scoring systems (APACHE III, SAPSII, and SOFA) significantly improves its predictive accuracy, and the results of NRI versus IDI were consistent with changes in the area under the curve. This research is the initial investigation to demonstrate a nonlinear, U-shaped connection between SHR and mortality in CS-AKI patients, identifying a critical risk threshold at SHR = 1.39. This finding suggests the potential decision-making value of SHR in patients with CS-AKI. In addition, the results of the subgroup analyses further supported the above findings, in which COPD resulted in a significantly stronger predictive validity for mortality risk (interaction *p* = 0.039). This finding suggests the need for clinically more stringent glycemic monitoring and interventions in patients with comorbid COPD to improve the adverse outcomes related to stress hyperglycemia.

It has been shown that perioperative glucose fluctuations increase the incidence of postoperative adverse events, especially in cardiac surgery, which not only increases the possibility of postoperative AKI, myocardial infarction, atrial fibrillation, infection, and other complications in patients but also increases the risk of mortality in the hospital after surgery [20,21]. However, conventional measurements of post-hospitalization glucose are unable to reflect transient changes in blood glucose levels [22]. The SHR captures the dynamic interplay between acute glucose dysregulation and the baseline metabolic state [23]. Cox proportional hazards models consistently demonstrated a significant correlation between elevated SHR and mortality risk in CS-AKI patients. In our study, multivariate Cox proportional hazards regression analyses showed that SHR quartile 4 (Q4: SHR ≥1.61) was independently associated with mortality in three models (Table 2). Furthermore, a body of research has demonstrated a robust correlation between SHR and patient outcome. Chen et al. [24] found in a retrospective cohort study that SHR quartiles Q3 and Q4 were independent risk factors for in-hospital mortality and intensive care unit mortality in patients with cerebrovascular disease, and their subgroup analyses showed that SHR was more pronounced in nondiabetic patients. Comparable outcomes were noted in the present study. In addition, a large retrospective study found that for patients undergoing cardiac surgery, hyperglycemia occurred postoperatively in 26% of nondiabetic patients, 46.5% of diabetes mellitus patients not treated with medication, 62.8% of oral hypoglycemic medication patients, and 73.8% of insulin-treated patients (*p* < 0.001) [25]. Stratified analysis showed that the short-term effects of SHR were stronger in non-diabetic patients. This outcome is consistent with previous studies that nondiabetic patients lack compensatory mechanisms for prolonged hyperglycemia and are more susceptible to damage from oxidative stress and inflammatory responses induced by acute glucose fluctuations (Supplementary eTable 8). Current research suggests that acute hyperglycemia can lead to NF-kB activation and production of inflammatory cytokines, resulting in impaired renal function [26–29]. Moreover, hyperglycemia can enhance the formation of advanced glycation end products (AGEs) and activate protein kinase C (PKC) through the polyol pathway and hexosamine pathway, thereby inducing excessive ROS production, exacerbating cellular oxidative stress and mitochondrial damage, and ultimately leading to renal cell injury [30,31]. Additionally, dysregulation of renal glucose metabolism activates pathways including nuclear factor kappa B (NF-κB) and TGF-β, leading to increased expression of inflammatory cytokines and markers such as TNF-α and interleukin-1β (IL-1β). This triggers an inflammatory response, resulting in renal injury and fibrosis [32,33]. Therefore, glycemic assessment in patients with CS-AKI is important. The study is the first finding of a U-shaped correlation between SHR and mortality for patients, which implies that controlling postoperative hyperglycemia alone may not be sufficient to change the prognosis and that dramatic fluctuations in stress blood glucose should be avoided. In a multicenter study, Ling et al. [34] reported that SHR was significantly correlated with the development of AKI, with both low SHR (< 0.7) and high SHR (≥1.1) linked to a substantial elevation in the risk of AKI (*p* < 0.001). In addition, in another large retrospective study, SHR had a U-shaped correlation with all-cause and cardiovascular mortality in patients, with the fourth quartile group of SHR demonstrating the greatest death rate [35], which is similar to our findings.

Subgroup analysis shows that the correlation between SHR and mortality in CS-AKI patients is stronger in non-diabetic patients, which may be related to the lack of an adaptive regulatory system for persistent hyperglycemia in non-diabetic patients. In contrast to diabetic patients, non-diabetic patients are more prone to significant glucose fluctuations during acute stress states, leading to more oxidative stress damage [36]. Additionally, subgroup analysis showed a major difference in the prognostic impact of SHR comparing COPD and non-COPD patients (P for interaction = 0.039). The results showed a risk ratio (HR = 7.18, 95% CI: 3.11–16.56) among patients with COPD, suggesting that COPD may worsen the negative effects of stress hyperglycemia. This effect could be related to the association of COPD patients with metabolic disorders, chronic inflammation, and oxidative stress, all of which may interact with hyperglycemia to worsen organ damage or disease progression. Additionally, patients undergoing CABG and combined procedures exhibited a significantly increased risk of mortality, whereas those undergoing valve surgery and other procedures showed no significant impact. There was no significant interaction between surgical type and SHR (*p* = 0.144), indicating that the overall risk trend for SHR was not altered by surgical type.

Furthermore, our study assessed the enhancement of mortality prediction in patients with CS-AKI by including SHR in established risk scores. The findings showed that the inclusion of SHR significantly improved the prediction accuracy for every assessed model. The SHR combined with the APACHE III score was the strongest predictor of patient outcomes in the new model developed (AUC = 0.766). The NRI and IDI further validated these improvements, highlighting the unique ability of the SHR to refine risk stratification in the perioperative period. This improvement in discrimination and reclassification indicates that SHR captures additional prognostic information beyond conventional risk indices, reflecting an individual’s metabolic response to surgical stress. In our study, an elevated SHR (Q4 ≥ 1.61) may serve as an early warning marker to identify high-risk patients after cardiac surgery. For such patients, clinicians could consider intensified perioperative glucose monitoring, tighter control of glycemic variability, optimization of hemodynamic status, and early nephrology consultation or lower thresholds for initiating renal replacement therapy. Importantly, integrating SHR into standard ICU risk assessment pathways may facilitate earlier recognition of vulnerable patients and more individualized management strategies. Although perioperative HbA1c measurement is not universally performed, it is increasingly routine in cardiac surgical practice, and the availability of point-of-care HbA1c assays could make real-time SHR calculation feasible. Currently, there are limited studies that have improved CS-AKI prognostic modeling by incorporating SHR. In a prognostic study, Lin et al. [37] integrated SHR into an existing risk prediction model, and the new model had better predictive performance. In addition, Chen et al. [24] found that incorporating SHR into existing models (APACHE III, SAPS II, and SOFA) resulted in an increase in the area under the curve as well as IDI and NRI.

This study has several strengths that enhance its clinical relevance. First, the large sample size (*n* = 3,249) derived from a well-validated database (MIMIC-IV) ensures robust statistical power and generalizability to critically ill populations. Second, the rigorous adjustment for confounders across three Cox regression models strengthens the independent prognostic value of SHR, including demographic, biochemical, and comorbidity-related variables. Third, our identification of a nonlinear U-shaped relationship between SHR and mortality provides novel insights into the dual risks of both excessive and insufficient stress glycemic responses, challenging the conventional focus on hyperglycemia alone. Additionally, the integration of SHR with established prognostic scores (APACHE III, SAPS II, SOFA) significantly improved mortality prediction, as evidenced by increased AUC, NRI, and IDI values, underscoring its incremental clinical utility. However, there are several limitations to consider. As a retrospective observational study, unmeasured residual confounding (e.g., undocumented glycemic variability, intraoperative factors, or medication use) may persist despite multivariable adjustments. In addition, the clinical application of SHR is dependent on the detection of HbA1c. HbA1c is not recorded in all patients in the MIMIC-IV database, and it is not a routine test in the clinical perioperative period, especially in patients without a history of diabetes mellitus, which limits the real-time application of SHR. Moreover, the MIMIC-IV database originates from a single center, which may affect external validity. Patients with ICU stays of less than 24 h were excluded from this study to ensure data completeness. However, this practice may reduce the extrapolation of the study results. The exclusion of this subset of patients, which includes rapid postoperative recoveries and early deaths, may introduce selection bias and preclude validation of the prognostic value of SHR in these extreme populations. Future studies should further explore this subgroup. Despite these limitations, our findings highlight SHR as a practical, dynamic biomarker for risk stratification in CS-AKI. Finally, a limitation of this study is the lack of detailed intraoperative parameters in the MIMIC-IV database, including use of extracorporeal circulation, duration of extracorporeal circulation, duration of aortic clamping, and type of procedure. These factors are known determinants of CS-AKI, and their absence may lead to residual confounders. Future prospective studies should validate these results and explore whether targeted glycemic interventions guided by SHR thresholds can mitigate mortality risks in this vulnerable population.

## Conclusion

In conclusion, this study emphasized the importance of SHR as a prognostic marker for patients, and the results showed a U-shaped association between SHR and death in patients with CS-AKI.

## Supplementary Material

Revised Supplementary.docx

## Data Availability

The MIMIC dataset is publicly available at https://physionet.org/content/mimiciv.
