# Supplementary material for: Nonlinear relationship between stress hyperglycemic ratio and prognosis in patients with cardiac surgery-related kidney injury: a retrospective cohort study
Source: Ren Fail. 2026 Jan 28;48(1):2613479. doi: 10.1080/0886022X.2026.2613479 (PMC12857695; doi:10.1080/0886022X.2026.2613479)

**eTable1. The ICD codes of cardiac surgeries and the comorbidities.**

| Cardiac surgeries | 3603,362,3610,3611,3612,3613,3614,3615,3616,3617,3619,021,0210083,0210088,0210089,021008C,021008F,021008W,0210093,0210098,0210099,021009C,021009F,021009W,02100A3,02100A8,02100A9,02100AC,02100AF,02100AW,02100J3,02100J8,02100J9,02100JC,02100JF,02100JW,02100K3,02100K8,02100K9,02100KC,02100KF,02100KW,02100Z3,02100Z8,02100Z9,02100ZC,02100ZF,0210344,02103D4,0210444,0210483,0210488,0210489,021048C,021048F,021048W,0210493,0210498,0210499,021049C,021049F,021049W,02104A3,02104A8,02104A9,02104AC,02104AF,02104AW,02104D4,02104J3,02104J8,02104J9,02104JC,02104JF,02104JW,02104K3,02104K8,02104K9,02104KC,02104KF,02104KW,02104Z3,02104Z8,02104Z9,02104ZC,02104ZF,0211083,0211088,0211089,021108C,021108F,021108W,0211093,0211098,0211099,021109C,021109F,021109W,02110A3,02110A8,02110A9,02110AC,02110AF,02110AW,02110J3,02110J8,02110J9,02110JC,02110JF,02110JW,02110K3,02110K8,02110K9,02110KC,02110KF,02110KW,02110Z3,02110Z8,02110Z9,02110ZC,02110ZF,0211344,02113D4,0211444,0211483,0211488,0211489,021148C,021148F,021148W,0211493,0211498,0211499,021149C,021149F,021149W,02114A3,02114A8,02114A9,02114AC,02114AF,02114AW,02114D4,02114J3,02114J8,02114J9,02114JC,02114JF,02114JW,02114K3,02114K8,02114K9,02114KC,02114KF,02114KW,02114Z3,02114Z8,02114Z9,02114ZC,02114ZF,0212083,0212088,0212089,021208C,021208F,021208W,0212093,0212098,0212099,021209C,021209F,021209W,02120A3,02120A8,02120A9,02120AC,02120AF,02120AW,02120J3,02120J8,02120J9,02120JC,02120JF,02120JW,02120K3,02120K8,02120K9,02120KC,02120KF,02120KW,02120Z3,02120Z8,02120Z9,02120ZC,02120ZF,0212344,02123D4,0212444,0212483,0212488,0212489,021248C,021248F,021248W,0212493,0212498,0212499,021249C,021249F,021249W,02124A3,02124A8,02124A9,02124AC,02124AF,02124AW,02124D4,02124J3,02124J8,02124J9,02124JC,02124JF,02124JW,02124K3,02124K8,02124K9,02124KC,02124KF,02124KW,02124Z3,02124Z8,02124Z9,02124ZC,02124ZF,0213083,0213088,0213089,021308C,021308F,021308W,0213093,0213098,0213099,021309C,021309F,021309W,02130A3,02130A8,02130A9,02130AC,02130AF,02130AW,02130J3,02130J8,02130J9,02130JC,02130JF,02130JW,02130K3,02130K8,02130K9,02130KC,02130KF,02130KW,02130Z3,02130Z8,02130Z9,02130ZC,02130ZF,0213344,02133D4,0213444,0213483,0213488,0213489,021348C,021348F,021348W,0213493,0213498,0213499,021349C,021349F,021349W,02134A3,02134A8,02134A9,02134AC,02134AF,02134AW,02134D4,02134J3,02134J8,02134J9,02134JC,02134JF,02134JW,02134K3,02134K8,02134K9,02134KC,02134KF,02134KW,02134Z3,02134Z8,02134Z9,02134ZC,02134ZF, 0203,024F07J,024F08J,024F0JJ,024F0KJ,024G072,024G082,024G0J2,024G0K2,024J072,024J082,024J0J2,024J0K2,025F0ZZ,025F3ZZ,025F4ZZ,025G0ZZ,025G3ZZ,025G4ZZ,025H0ZZ,025H3ZZ,025H4ZZ,025J0ZZ,025J3ZZ,025J4ZZ,027F04Z,027F0DZ,027F0ZZ,027F34Z,027F3DZ,027F3ZZ,027F44Z,027F4DZ,027F4ZZ,027G04Z,027G0DZ,027G0ZZ,027G34Z,027G3DZ,027G3ZZ,027G44Z,027G4DZ,027G4ZZ,027H04Z,027H0DZ,027H0ZZ,027H34Z,027H3DZ,027H3ZZ,027H44Z,027H4DZ,027H4ZZ,027J04Z,027J0DZ,027J0ZZ,027J34Z,027J3DZ,027J3ZZ,027J44Z,027J4DZ,027J4ZZ,02BF0ZX,02BF0ZZ,02BF3ZX,02BF3ZZ,02BF4ZX,02BF4ZZ,02BG0ZX,02BG0ZZ,02BG3ZX,02BG3ZZ,02BG4ZX,02BG4ZZ,02BH0ZX,02BH0ZZ,02BH3ZX,02BH3ZZ,02BH4ZX,02BH4ZZ,02BJ0ZX,02BJ0ZZ,02BJ3ZX,02BJ3ZZ,02BJ4ZX,02BJ4ZZ,02CF0ZZ,02CF3ZZ,02CF4ZZ,02CG0ZZ,02CG3ZZ,02CG4ZZ,02CH0ZZ,02CH3ZZ,02CH4ZZ,02CJ0ZZ,02CJ3ZZ,02CJ4ZZ,02LH0CZ,02LH0DZ,02LH0ZZ,02LH3CZ,02LH3DZ,02LH3ZZ,02LH4CZ,02LH4DZ,02LH4ZZ,02NF0ZZ,02NF3ZZ,02NF4ZZ,02NG0ZZ,02NG3ZZ,02NG4ZZ,02NH0ZZ,02NH3ZZ,02NH4ZZ,02NJ0ZZ,02NJ3ZZ,02NJ4ZZ,02QF0ZJ,02QF0ZZ,02QF3ZJ,02QF3ZZ,02QF4ZJ,02QF4ZZ,02QG0ZE,02QG0ZZ,02QG3ZE,02QG3ZZ,02QG4ZE,02QG4ZZ,02QH0ZZ,02QH3ZZ,02QH4ZZ,02QJ0ZG,02QJ0ZZ,02QJ3ZG,02QJ3ZZ,02QJ4ZG,02QJ4ZZ,02RF07Z,02RF08N,02RF08Z,02RF0JZ,02RF0KZ,02RF37H,02RF37Z,02RF38H,02RF38N,02RF38Z,02RF3JH,02RF3JZ,02RF3KH,02RF3KZ,02RF47Z,02RF48N,02RF48Z,02RF4JZ,02RF4KZ,02RG07Z,02RG08Z,02RG0JZ,02RG0KZ,02RG37H,02RG37Z,02RG38H,02RG38Z,02RG3JH,02RG3JZ,02RG3KH,02RG3KZ,02RG47Z,02RG48Z,02RG4JZ,02RG4KZ,02RH07Z,02RH08Z,02RH0JZ,02RH0KZ,02RH37H,02RH37Z,02RH38H,02RH38L,02RH38M,02RH38Z,02RH3JH,02RH3JZ,02RH3KH,02RH3KZ,02RH47Z,02RH48Z,02RH4JZ,02RH4KZ,02RJ07Z,02RJ08Z,02RJ0JZ,02RJ0KZ,02RJ37H,02RJ37Z,02RJ38H,02RJ38Z,02RJ3JH,02RJ3JZ,02RJ3KH,02RJ3KZ,02RJ47Z,02RJ48Z,02RJ4JZ,02RJ4KZ,02TH0ZZ,02TH3ZZ,02TH4ZZ,02UF07J,02UF07Z,02UF08J,02UF08Z,02UF0JJ,02UF0JZ,02UF0KJ,02UF0KZ,02UF37J,02UF37Z,02UF38J,02UF38Z,02UF3JJ,02UF3JZ,02UF3KJ,02UF3KZ,02UF47J,02UF47Z,02UF48J,02UF48Z,02UF4JJ,02UF4JZ,02UF4KJ,02UF4KZ,02UG07E,02UG07Z,02UG08E,02UG08Z,02UG0JE,02UG0JZ,02UG0KE,02UG0KZ,02UG37E,02UG37Z,02UG38E,02UG38Z,02UG3JE,02UG3JH,02UG3JZ,02UG3KE,02UG3KZ,02UG47E,02UG47Z,02UG48E,02UG48Z,02UG4JE,02UG4JZ,02UG4KE,02UG4KZ,02UH07Z,02UH08Z,02UH0JZ,02UH0KZ,02UH37Z,02UH38Z,02UH3JZ,02UH3KZ,02UH47Z,02UH48Z,02UH4JZ,02UH4KZ,02UJ07G,02UJ07Z,02UJ08G,02UJ08Z,02UJ0JG,02UJ0JZ,02UJ0KG,02UJ0KZ,02UJ37G,02UJ37Z,02UJ38G,02UJ38Z,02UJ3JG,02UJ3JZ,02UJ3KG,02UJ3KZ,02UJ47G,02UJ47Z,02UJ48G,02UJ48Z,02UJ4JG,02UJ4JZ,02UJ4KG,02UJ4KZ,02VG0ZZ,02VG3ZZ,02VG4ZZ,02WF07Z,02WF08Z,02WF0JZ,02WF0KZ,02WF37Z,02WF38Z,02WF3JZ,02WF3KZ,02WF47Z,02WF48Z,02WF4JZ,02WF4KZ,02WG07Z,02WG08Z,02WG0JZ,02WG0KZ,02WG37Z,02WG38Z,02WG3JZ,02WG3KZ,02WG47Z,02WG48Z,02WG4JZ,02WG4KZ,02WH07Z,02WH08Z,02WH0JZ,02WH0KZ,02WH37Z,02WH38Z,02WH3JZ,02WH3KZ,02WH47Z,02WH48Z,02WH4JZ,02WH4KZ,02WJ07Z,02WJ08Z,02WJ0JZ,02WJ0KZ,02WJ37Z,02WJ38Z,02WJ3JZ,02WJ3KZ,02WJ47Z,02WJ48Z,02WJ4JZ,02WJ4KZ,3500,3501,3502,3503,3504,3505,3506,3507,3508,3509,3510,3511,3512,3513,3514,3520,3521,3522,3523,3524,3525,3526,3527,3528,3533,3539,3552,3596,3597,3599,X2RF032,X2RF332 |
| --- | --- |

**eTable 2. Distribution of Acute Kidney Injury Following Cardiac Surgery**

| Variables | Total (n = 3249) | 1 (n = 812) | 2 (n = 811) | 3 (n = 813) | 4 (n = 813) | Statistic | *P* |
| --- | --- | --- | --- | --- | --- | --- | --- |
|  |  |  |  |  |  |  |  |
| Aki Stage, n (%) |  |  |  |  |  | χ²=25.80 | **<.001** |
| 1 | 2515 (77.41) | 614 (75.62) | 649 (80.02) | 640 (78.72) | 612 (75.28) |  |  |
| 2 | 622 (19.14) | 171 (21.06) | 141 (17.39) | 157 (19.31) | 153 (18.82) |  |  |
| 3 | 112 (3.45) | 27 (3.33) | 21 (2.59) | 16 (1.97) | 48 (5.90) |  |  |
| χ²: Chi-square test | | | | | | | |

**Table 3. Outcomes for patients with BMI data**

| Clinical outcomes | Total (n = 152) | Q1 (n = 33) | Q (n = 38) | Q3 (n = 43) | Q4 (n = 38) | P |
| --- | --- | --- | --- | --- | --- | --- |
|  |  |  |  |  |  | 0.501 |
| 30-day mortality, (%) | 3 (1.97) | 0 (0.00) | 0 (0.00) | 1 (2.33) | 2 (5.26) |  |
|  |  |  |  |  |  | 0.496 |
| 30-day mortality, (%) | 6 (3.95) | 0 (0.00) | 1 (2.63) | 2 (4.65) | 3 (7.89) |  |
|  |  |  |  |  |  | 0.836 |
| 30-day mortality, (%) | 14 (9.21) | 3 (9.09) | 3 (7.89) | 3 (6.98) | 5 (13.16) |  |

**eTable 4. Table of Baseline Characteristics for Patients with BMI Data**

| Variables | Total (n = 152) | 1 (n = 33) | 2 (n = 38) | 3 (n = 43) | 4 (n = 38) | *P* |
| --- | --- | --- | --- | --- | --- | --- |
|  |  |  |  |  |  |  |
| HbA1c | 5.90 (5.50, 6.70) | 7.30 (6.50,8.70) | 6.00 (5.70,6.68) | 5.80 (5.50,6.10) | 5.50 (5.20,5.80) | **<.001** |
| Glucose | 176.50 (158.00, 200.00) | 166.00 (148.00,190.00) | 161.50 (153.25,184.25) | 176.00 (165.50,193.50) | 202.50 (183.25,225.00) | **<.001** |
| SHR | 1.42 (1.23, 1.61) | 1.08 (0.81,1.14) | 1.27 (1.23,1.35) | 1.50 (1.44,1.55) | 1.80 (1.70,1.96) | **<.001** |
| WBC | 15.95 (12.78, 19.47) | 16.20 (11.40,19.00) | 14.40 (11.25,19.12) | 16.20 (13.35,19.45) | 16.15 (13.85,19.62) | 0.316 |
| Creatinine | 0.90 (0.70, 1.10) | 0.90 (0.80,1.10) | 0.85 (0.70,1.00) | 0.90 (0.70,1.20) | 0.90 (0.80,1.10) | 0.632 |
| BUN | 16.00 (13.00, 22.00) | 17.00 (12.00,23.00) | 16.00 (13.25,20.00) | 15.00 (12.00,21.50) | 16.50 (13.25,22.00) | 0.910 |
| Platelet | 149.00 (113.75, 175.25) | 158.00 (136.00,175.00) | 136.50 (117.00,161.75) | 152.00 (113.50,177.50) | 135.00 (106.75,175.50) | 0.358 |
| Sodium | 135.00 (134.00, 137.00) | 135.00 (134.00,136.00) | 135.00 (134.00,136.75) | 137.00 (133.00,138.00) | 136.00 (134.00,137.00) | 0.292 |
| Potassium | 4.60 (4.10, 5.00) | 4.50 (4.20,4.90) | 4.60 (4.10,5.05) | 4.60 (4.05,5.00) | 4.70 (4.10,5.10) | 0.924 |
| Hemoglobin | 9.70 (8.20, 11.35) | 8.90 (7.75,11.05) | 9.50 (8.20,11.10) | 10.10 (8.90,11.40) | 10.20 (8.10,11.80) | 0.524 |
| Weight | 90.90 (80.68, 106.32) | 95.00 (82.30,105.30) | 89.55 (80.95,107.05) | 89.80 (80.25,104.75) | 96.80 (80.75,106.97) | 0.890 |
| Height | 173.00 (165.00, 180.00) | 168.00 (163.00,178.00) | 174.00 (165.75,180.00) | 173.00 (168.00,178.00) | 175.00 (165.75,180.00) | 0.845 |
| Heart Rate | 80.00 (75.75, 87.00) | 80.00 (68.00,85.00) | 80.00 (75.25,87.75) | 80.00 (76.50,85.50) | 80.00 (80.00,88.00) | 0.571 |
| SBP | 113.00 (101.75, 123.25) | 114.00 (101.00,128.00) | 113.00 (102.50,119.00) | 116.00 (103.00,125.00) | 112.00 (100.00,123.75) | 0.880 |
| DBP | 58.00 (52.00, 64.00) | 57.00 (51.00,63.00) | 59.00 (52.00,62.00) | 58.00 (52.50,64.00) | 58.50 (53.25,64.75) | 0.953 |
| Resp Rate | 15.75 (14.00, 17.00) | 15.00 (14.00,16.00) | 14.50 (14.00,16.00) | 16.00 (14.00,17.50) | 16.00 (15.00,18.00) | 0.062 |
| SAPS II | 36.00 (31.00, 43.25) | 35.00 (31.00,45.00) | 35.00 (31.00,40.00) | 36.00 (30.00,46.50) | 37.00 (32.00,42.25) | 0.917 |
| APACHE III | 35.00 (28.00, 48.00) | 35.00 (32.00,48.00) | 33.00 (25.25,40.75) | 38.00 (26.50,50.50) | 35.00 (30.00,44.75) | 0.275 |
| SOFA | 3.00 (1.00, 4.00) | 2.00 (1.00,4.00) | 3.00 (1.00,4.00) | 2.00 (1.00,4.00) | 3.50 (2.00,5.00) | 0.436 |
| BMI | 30.70 (27.96, 34.17) | 32.28 (28.44,34.01) | 29.67 (28.05,34.95) | 29.32 (27.43,32.80) | 31.35 (28.45,34.39) | 0.490 |

**Note:** HbA1c, glycosylated hemoglobin; SHR, the stress-induced hyperglycemia ratio; WBC, white blood cell; BUN, blood urea nitrogen; SBP, systolic blood pressure; DBP, diastolic blood pressure; SAPS II, Simplified Acute Physiology Score; APACHE III, Acute Physiology and Chronic Health Evaluation III; SOFA, Sequential Organ Failure Assessment; BMI, body mass index

**Table 5. The Variance inflation factors (VIFs) of each covariate**

|  | Tolerance | VIF |
| --- | --- | --- |
| WBC | 0.8477706 | 1.1795644 |
| BUN | 0.564716 | 1.7708016 |
| Platelet | 0.8491173 | 1.1776937 |
| Sodium | 0.9853582 | 1.0148594 |
| SBP | 0.6161443 | 1.6229964 |
| DBP | 0.5679605 | 1.7606858 |
| Creatinine | 0.5976813 | 1.6731324 |
| Potassium | 0.8678665 | 1.152251 |
| Hemoglobin | 0.7847007 | 1.2743712 |
| Resp rate | 0.9118923 | 1.0966208 |
| Heart rate | 0.8430399 | 1.1861835 |
| SOFA | 0.6711954 | 1.4898792 |
| APACHE III | 0.369735 | 2.7046398 |
| SAPS II | 0.3835981 | 2.6068954 |
| Myocardial infarct | 0.9219844 | 1.0846171 |
| Congestive heart failure | 0.8470493 | 1.1805689 |
| Chronic pulmonary disease | 0.9423174 | 1.0612136 |
| Malignant cancer | 0.9624883 | 1.0389737 |
| Diabetes mellitus | 0.902184 | 1.1084213 |

**Note:** WBC, white blood cell; BUN, blood urea nitrogen; SBP, systolic blood pressure; DBP, diastolic blood pressure; APACHE III, Acute Physiology and Chronic Health Evaluation III; SAPS II, Simplified Acute Physiology Score; SOFA, Sequential Organ Failure Assessment

**eTable 6.** **Analysis of the interaction between SHR and diabetes mellitus**

| Variable | outcome | HR | 95%CI | P for Interaction (SHR×Diabetic) |
| --- | --- | --- | --- | --- |
| SHR |  |  |  |  |
|  | 30-day mortality | 1.643 | 1.228-2.197 | <.001 |
|  | 90-day mortality | 1.407 | 1.107-1.790 | 0.005 |
|  | 360-day mortality | 1.391 | 1.159-1.671 | <.001 |

**Note:** SHR, the stress-induced hyperglycemia ratio

**eTable 7. Multivariate Cox regression of diabetes mellitus component strata**

| Variable |  | Model 1 | | |  | Model 2 | | |  | Model 3 | | |
| --- | --- | --- | --- | --- | --- | --- | --- | --- | --- | --- | --- | --- |
|  |  | HR | 95%CI | P-value |  | HR | 95%CI | P-value |  | HR | 95%CI | P-value |
| SHR quartiles |  |  |  |  |  |  |  |  |  |  |  |  |
|  | 30-day mortality | | |  |  |  |  |  |  |  |  |  |
|  | Q1 | 1 |  |  |  | 1 |  |  |  | 1 |  |  |
|  | Q2 | 2.14 | 0.94 - 4.88 | 0.069 |  | 1.83 | 0.78 - 4.25 | 0.162 |  | 1.81 | 0.78 - 4.21 | 0.171 |
|  | Q3 | 2.76 | 1.20 - 6.32 | 0.016 |  | 2.25 | 0.96 - 5.30 | 0.062 |  | 2.31 | 0.98 - 5.47 | 0.057 |
|  | Q4 | 2.99 | 1.59 - 7.39 | 0.002 |  | 2.96 | 1.26 - 6.97 | 0.013 |  | 2.94 | 1.27 - 7.06 | 0.012 |
|  | 90-day mortality |  |  |  |  |  |  |  |  |  |  |  |
|  | Q1 | 1 |  |  |  | 1 |  |  |  | 1 |  |  |
|  | Q2 | 1.98 | 1.00 - 3.93 | 0.052 |  | 1.85 | 0.91 - 3.75 | 0.091 |  | 1.87 | 0.92 - 3.82 | 0.084 |
|  | Q3 | 2.5 | 1.25 - 4.99 | 0.009 |  | 2.29 | 1.12 - 4.69 | 0.024 |  | 2.22 | 1.08 - 4.59 | 0.031 |
|  | Q4 | 2.9 | 1.42 - 5.92 | 0.004 |  | 2.59 | 1.23 - 5.47 | 0.012 |  | 2.54 | 1.20 - 5.36 | 0.015 |
|  | 360-day mortality |  |  |  |  |  |  |  |  |  |  |  |
|  | Q1 | 1 |  |  |  | 1 |  |  |  | 1 |  |  |
|  | Q2 | 1.08 | 0.65 - 1.80 | 0.757 |  | 1.03 | 0.61 - 1.75 | 0.898 |  | 1.07 | 0.63 - 1.81 | 0.809 |
|  | Q3 | 1.4 | 0.84 - 2.33 | 0.196 |  | 1.24 | 0.73 - 2.13 | 0.426 |  | 1.21 | 0.7 - 2.08 | 0.495 |
|  | Q4 | 1.95 | 1.18 – 3.23 | 0.009 |  | 1.77 | 1.05 – 2.99 | 0.032 |  | 1.71 | 1.01 – 2.88 | 0.045 |
| Inclusion variables: model 1: Sex, Age, Systolic blood pressure, Heart rate, Respiratory rate; model 2: model 1 + White blood cell count, Platelet count, Creatinine, Blood urea nitrogen, Hemoglobin, Serum sodium, Serum potassium model 3: model 2 + Malignancy, Congestive heart failure, COPD, Diabetes mellitus, Acute myocardial infarction | | | | | | | | | | | | |

**eTable 8. Multivariate Cox Regression for Non-Diabetic Component Strata**

| Variable |  | Model 1 | | |  | Model 2 | | |  | Model 3 | | |
| --- | --- | --- | --- | --- | --- | --- | --- | --- | --- | --- | --- | --- |
|  |  | HR | 95%CI | P-value |  | HR | 95%CI | P-value |  | HR | 95%CI | P-value |
| SHR quartiles |  |  |  |  |  |  |  |  |  |  |  |  |
|  | 30-day mortality |  |  |  |  |  |  |  |  |  |  |  |
|  | Q1 | 1 |  |  |  | 1 |  |  |  | 1 |  |  |
|  | Q2 | 0.54 | 0.13 - 2.16 | 0.382 |  | 0.49 | 0.12 - 2.01 | 0.321 |  | 0.59 | 0.14 - 2.44 | 0.466 |
|  | Q3 | 0.67 | 0.19 - 2.37 | 0.531 |  | 0.57 | 0.15 - 2.16 | 0.41 |  | 0.62 | 0.16 - 2.37 | 0.486 |
|  | Q4 | 3.14 | 1.07 - 8.27 | 0.047 |  | 3.02 | 1.02 - 9.00 | 0.047 |  | 3.3 | 1.06 - 9.94 | 0.034 |
|  | 90-day mortality |  |  |  |  |  |  |  |  |  |  |  |
|  | Q1 | 1 |  |  |  | 1 |  |  |  | 1 |  |  |
|  | Q2 | 0.56 | 0.22 - 1.46 | 0.239 |  | 0.66 | 0.23 - 1.89 | 0.443 |  | 0.75 | 0.26 - 2.14 | 0.589 |
|  | Q3 | 0.64 | 0.26 - 1.56 | 0.324 |  | 0.72 | 0.26 - 1.97 | 0.525 |  | 0.79 | 0.29 - 2.17 | 0.647 |
|  | Q4 | 2.12 | 1.00 - 4.52 | 0.051 |  | 2.31 | 0.95 - 5.60 | 0.065 |  | 2.53 | 1.03 - 6.21 | 0.042 |
|  | 360-day mortality |  |  |  |  |  |  |  |  |  |  |  |
|  | Q1 | 1 |  |  |  | 1 |  |  |  | 1 |  |  |
|  | Q2 | 0.7 | 0.37 - 1.33 | 0.279 |  | 0.73 | 0.37 - 1.43 | 0.354 |  | 0.8 | 0.41 - 1.59 | 0.529 |
|  | Q3 | 0.63 | 0.33 - 1.19 | 0.151 |  | 0.66 | 0.34 - 1.30 | 0.229 |  | 0.71 | 0.36 - 1.40 | 0.325 |
|  | Q4 | 1.43 | 0.82 – 2.50 | 0.203 |  | 1.39 | 0.75 – 2.55 | 0.295 |  | 1.5 | 0.81 – 2.79 | 0.195 |
| Inclusion variables: model 1: Sex, Age, Systolic blood pressure, Heart rate, Respiratory rate; model 2: model 1 + White blood cell count, Platelet count, Creatinine, Blood urea nitrogen, Hemoglobin, Serum sodium, Serum potassium model 3: model 2 + Malignancy, Congestive heart failure, COPD, Diabetes mellitus, Acute myocardial infarction | | | | | | | | | | | | |

eFig.1 30-day APACHE III+SHR


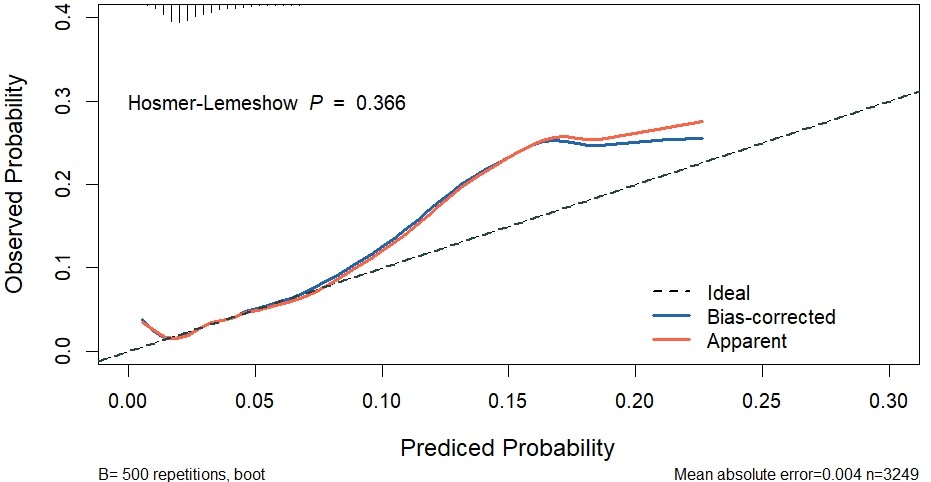


eFig.2 30-day SAPSII+SHR


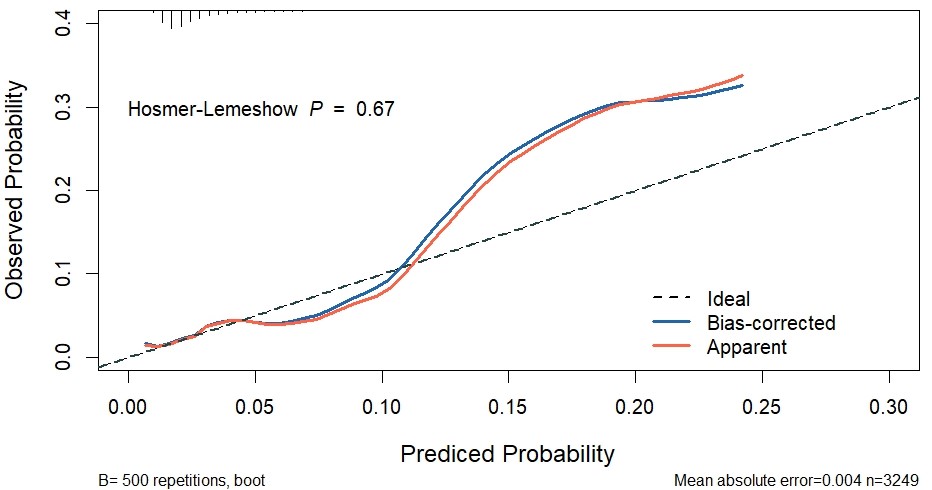


eFig.3 30-day SOFA+SHR


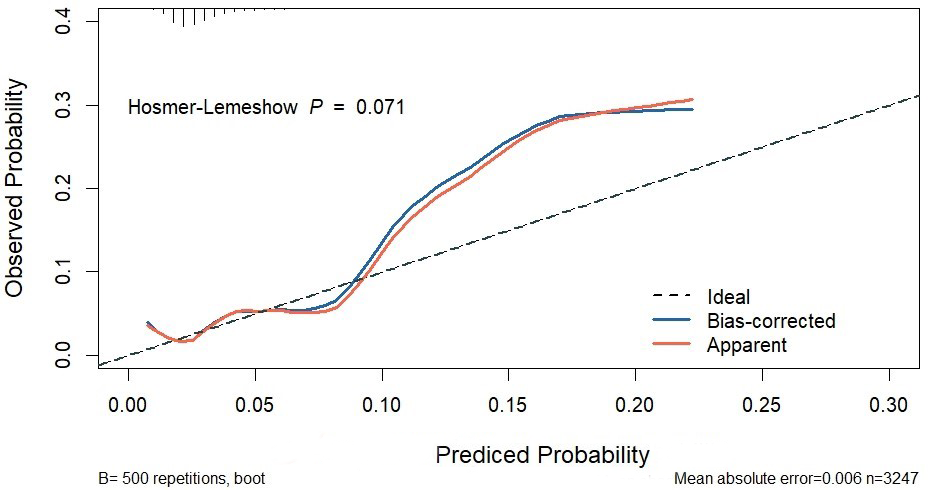

Supplement: Revised Supplementary.docx [file IRNF_A_2613479_SM7175.docx]
